# Evaluation of the mutant selection window of danofloxacin against *Actinobacillus pleuropneumoniae* in an *in vitro* dynamic model

**DOI:** 10.3389/fvets.2023.1107608

**Published:** 2023-01-30

**Authors:** Longfei Zhang, Hongjuan Wang, Yilin Bai, Lei Wang, Yueyu Bai, Jianhe Hu

**Affiliations:** ^1^College of Animal Science and Veterinary Medicine of Henan Institute of Science and Technology, Xinxiang, China; ^2^School of Agricultural Sciences, Zhengzhou University, Zhengzhou, China

**Keywords:** multidrug resistance, peristaltic pump, PK/PD, mutation selection window, *Actinobacillus pleuropneumoniae*

## Abstract

**Introduction:**

The rapid emergence and widespread spread of multidrug-resistant bacteria is a serious threat to the health of humans and animals. The pharmacokinetic/pharmacodynamic (PK/PD) integration model based on mutant selection window (MSW) theory is an important method to optimize the dosage regimen to prevent the emergence and spread of drug-resistant bacteria. *Actinobacillus pleuropneumoniae* (AP) is a pathogen that can cause pleuropneumonia in pigs.

**Methods:**

We employed an *in vitro* dynamic infection model (DIM) to study the prevention of drug-resistant mutations of danofloxacin against AP. A peristaltic pump was applied to establish an *in vitro* DIM to simulate the PK of danofloxacin in plasma, and to study the MSW of danofloxacin against AP. A peristaltic-pump *in vitro* infection model was established to simulate dynamic changes in the danofloxacin concentration in pig plasma. PK and PD data were obtained. Then, the relationship between PK/PD parameters and antibacterial activity was analyzed by the sigmoid E_max_ model.

**Results and discussion:**

The area under the curve during 24 h/ the minimum concentration that inhibits colony formation by 99% (AUC_24h_/MIC_99_) had the best-fitting relationship with antibacterial activity. The AUC_24h_/MIC_99_ values for a bacteriostatic effect, bactericidal effect, and eradication effect were 2.68, 33.67, and 71.58 h, respectively. We hope these results can provide valuable guidance when using danofloxacin to treat AP infection.

## Introduction

*Actinobacillus pleuropneumoniae* (AP) is a pathogen that can cause pleuropneumonia in pigs. The clinical symptoms are fibrinous hemorrhagic pneumonia and necrotizing pneumonia (1–3). AP infection seriously affects development of the pig industry, and can result in considerable economic losses for farmers (4–7). The common prevention and treatment methods for AP infection are vaccination and drug therapy.

Vaccination is an efficacious means for preventing AP infection (8–10). However, the number of serotypes is large and the cross-protection of each serotype against AP is poor. Hence, developing a universal, stable vaccine that works on all serotypes is very difficult (11, 12). Antimicrobial therapy remains an efficacious way to treat AP infection.

The antibiotics used most commonly to treat AP infection in pigs are ceftiofur, tiamulin, danofloxacin, florfenicol, tilmicosin, and cefquinome. However, non-rational use of antimicrobial agents can result in the emergence and spread of drug-resistant bacteria, which leads to treatment failure (13–16). Development of new antibiotics and optimization of dosage regimens can be employed to address drug-resistance issues. The development of new drugs is time-consuming and cannot keep pace with the rate of bacterial mutations. Hence, optimization of dosage regimens can help to prevent the emergence of drug-resistant bacteria. The pharmacokinetics/pharmacodynamics (PK/PD) integration model based on mutant selection window (MSW) theory is an effective method to optimize dosage regimens to prevent drug resistance.

Dong et al. (17) were the first to propose that the mutant prevention concentration (MPC) is a limitation of the MSW theory. The MPC is defined as the lowest drug concentration that inhibits the growth of insensitive bacterial subpopulations at high bacterial concentrations (bacterial number ≥10^9^ CFU/mL). The minimal inhibitory concentration (MIC) is located in the lower part of the MSW. If the drug concentration is within the MSW (particularly in the lower–middle part of the MSW) and subject to multiple selective pressures, then resistant bacteria are selected over susceptible bacteria (18, 19).

The *in vitro* dynamic infection model (DIM) is convenient, economic, easy to operate, and can simulate PK and PD in infected target organs. It has important application value in optimizing drug-administration regimens for preventing drug-resistant mutations (20–24). The peristaltic pump is a commonly used *in vitro* model that can simulate the dynamic changes of drug concentrations and bacteria counts *in vivo*. This model can be employed to obtain the real-time and continuous antibacterial effect between a drug and bacteria.

Danofloxacin is a third-generation fluoroquinolone used only in animals. PK/PD studies have been carried out *in vivo* and *ex vivo* using danofloxacin. However, danofloxacin has not been studied *in vitro* to obtain real-time and continuous antibacterial concentrations.

Here, a peristaltic-pump model was employed to establish an *in vitro* infection model to study the prevention of drug-resistant mutations based on the MSW. Our results could provide valuable guidance for formulating dosage regimens if using danofloxacin to treat AP infection in clinical settings to prevent the emergence of drug-resistant mutations.

## Materials and methods

### Strains, drugs, and instruments

AP (CVCC259) was purchased from Chinese Veterinary Culture Collection Center (Qingdao, China). Danofloxacin mesylate powder (content >99%) was provided by Guangdong Dahuanong Biotechnology (Guangdong, China). Tryptic soy broth (TSB) and Mueller–Hinton Agar (MHA) were obtained from Guangdong Huankai Microbiology Technology (Guangdong, China). Nicotinamide adenine dinucleotide (NAD) was sourced from Beijing Puboxin Biotechnology (Beijing, China). Newborn bovine serum was provided by Guangzhou Ruite (Guangzhou, China).

A peristaltic pump (BT100-1F), pump head (DG-2-B/D; 10 roller), and rubber hose (inner diameter ≤3.17 mm; wall thickness = 0.8–1 mm) were purchased from Longer Precision Pump (Baoding, NC, USA). Fiber dialysis tubes (Float-A-Lyzer^®^ 1,000 kD; 10 mL) were sourced from MilliporeSigma (Burlington, MA, USA).

### Determination of the MIC, MIC_99_ and MPC

MHA and TSB were supplemented with 4% newborn bovine serum and NAD (1 mg/mL).

The MIC was tested by an agar-dilution method according to criteria set by the Clinical and Laboratory Standards Institute (25). Briefly, after being cultured for 8 h in a constant-temperature shaker (180–200 rpm, 37°C), the bacterial suspension was diluted to 10^6^ CFU/mL by TSB. Then, the bacterial suspension (100 μL) was added to an MHA plate containing danofloxacin (0.016–1 μg/mL after twofold dilution). After drying, the MHA plates were placed in an incubator in an atmosphere of 5% CO_2_ for 18–20 h at 37°C. The MIC was determined as the minimum concentration of drug that did not result in bacterial growth.

Next, we determined MIC_99_. Briefly, a series of MHA plates containing drugs were prepared based on the MIC (90% × MIC, 80% × MIC, 70% × MIC, 60% × MIC, 50% × MIC). After the logarithmic-phase bacterial suspension had been diluted tenfold (10^−1^, 10^−2^, 10^−3^, 10^−4^, 10^−5^, 10^−6^), the dilutions were inoculated to MHA and cultured as described for determination of the MIC. Then, the bacterial populations were counted and compared between drug-containing plates and the blank plate. Percent recovery growth of bacteria was obtained, and a linear formula between the drug concentration and percent recovery was obtained. MIC_99_ was determined as the value which inhibited the growth of bacteria by 99% (1% recovery).

We also tested the MPC. Briefly, after being cultured for 8 h, a logarithmic-phase bacterial suspension (100 mL) was centrifuged (5,000 × *g*, 20 min, 4°C). Then, the supernatant was removed and blank TSB (1 mL) was added for a bacterial population of 1.5 × 10^11^ CFU/mL. Then, the bacterial solution (100 μL) was inoculated on MHA plates (1 × MIC, 2 × MIC, 4 × MIC, 8 × MIC, 16 × MIC, 32 × MIC, 64 × MIC) and incubation allowed to proceed for 72 h. The minimum concentration of danofloxacin that did not elicit bacterial growth was defined as MPC_pr_. Then, based on MPC_pr_, the drug concentration was reduced linearly from 10%MPC_pr_ to 50% × MPC_pr_, and the procedure repeated as described for measurement of MPC_pr_. The MPC was defined as the lowest concentration of danofloxacin that could inhibit the growth of bacteria. All tests were repeated thrice.

### Establishment of an *in vitro* DIM

The peristaltic pump that we employed has been described in detail previously (26). A storage chamber, central chamber, and elimination chamber were connected through the peristaltic pump and rubber tube. The storage chamber consisted of a blue-cap bottle (500–5,000 mL) for storage of blank TSB broth. The central chamber comprised a modified three-necked flask containing blank TSB broth (290 mL), a dialysis tube, and magnetic rotor. The three-necked flask consisted of an inlet tube, sampling tube, and outlet tube with rubber stoppers. The sampling tube comprised an elongated syringe needle and nylon filters (0.22 μm) for collection of the TSB sample and contamination prevention. The central chamber was placed in a large beaker with water at a constant temperature (37°C) and magnetic-stirring apparatus (100 rpm). The elimination chamber consisted of a blue-cap bottle (500–5,000 mL) for collection of waste liquid. The dialysis tube contained a bacterial suspension (10 mL) and “floated” in blank TSB and 1-cm above TSB thanks to a foam gasket.

The PK parameter of danofloxacin in pigs was in reference to the work of Yang et al. (27). We set the elimination half-life (t_1/2_) of danofloxacin at 7 h. The elimination rate constant (Kel) was calculated to be 0.693/t_1/2_. The flow rate of the peristaltic pump (Q) was calculated as Kel × V_C_ (broth volume in the central chamber and dialysis tube). After the flow rate had been set, the device was run for 2 h to enable stabilization. Then, logarithmic-phase AP (10^8^ CFU/mL) was added to the central chamber. The *in vitro* DIM was established if the bacterial population stabilized at ~10^8^ CFU/mL.

### Kill curves and changes in the MIC

We wished to study the antibacterial effect in different parts of the MSW. Hence, seven dosage groups (0 × MIC_99_, 1/2 × MIC_99_, 1 × MIC_99_, 2 × MIC_99_, 4 × MIC_99_, 8 × MIC_99_, 16 × MIC_99_) were set up and administrated thrice every 24 h. To balance the drug concentration between the dialysis tube and peripheral chambers rapidly, both compartments were administered drugs to ensure that the drug concentration was identical upon experiment initiation. The bacterial suspension (0.1 mL) was collected from the dialysis chamber with a 1-mL sterile syringe. Then, it was diluted and dropped onto a blank MHA plate for bacterial counting at 0, 3, 6, 9, 12, and 24 h after each dose as well as at 48 and 72 h after the final dose. The limit of detection of the bacterial count was 50 CFU/mL. Each dose was repeated thrice. The kill curve of danofloxacin against AP was drawn as the logarithmic value of the bacterial population at different times.

To detect AP mutants, each sample was plated in MHA containing 1 × MIC of danofloxacin 24 h after each dose as well as 48 and 72 h after the final dose. AP with increasing MICs was passed through five generations in MHA to monitor the stability of the mutant. Then, the MIC of mutant AP was tested as described above.

### PK/PD fitting and analysis

The concentration of danofloxacin at different time points was tested by high-performance liquid chromatography, but the data were lost because of damage to software. Therefore, the PK of drugs in the model were simulated using a first-order elimination rate and calculated using Equation 1:


(1) $$\begin{array}{l} C=C_{0}\times e^{-kt} \end{array}$$

C: drug concentration at time t,

C_0_: initial concentration of danofloxacin,

K: constant of elimination rate,

t: time of sample collection after drug administration.

The drug concentration at each time point after each dose administration was calculated, and drug concentration–time curves were drawn. Values of area under concentration-time curve (AUC_24h_) and maximum concentration (C_max_) during 24 h were obtained based on a non-compartment model using WinNonlin (version 5.2.1, Pharsight, MO, USA).

The antibacterial effect (E) was defined as the maximum change in the number pf bacteria during the interval of each administration. The antibacterial effect was split into a bacteriostatic effect (0 log_10_ CFU/mL), bactericidal effect (3 log_10_ CFU/mL), and eradication effect (4 log_10_ CFU/mL).

AUC_24h_/MIC_99_ and C_max_/MIC_99_ were obtained directly by the values of AUC_24h_ and C_max_ divided by MIC_99_. The percentage of time that the drug concentration was above MIC_99_ during the dosing interval of 24 h (i.e., %T >MIC_99_) was calculated by PD models using WinNonlin.

The relationship between PK/PD parameters and the antibacterial effect was fitted by an inhibitory sigmoid E_max_ model by WinNonlin using Equation 2:


(2) $$\begin{array}{l} E=E_{\max}-\frac{\left(E_{\max}-E_{0}\right)\times C_{e}^{N}}{C_{e}^{N}+EC_{50}^{N}} \end{array}$$

E: change in the bacterial count in different drug concentrations after administration of each dose,

E_max_: change in the bacterial count in the control group after administration of each dose,

E_0_: maximum change in the bacterial count in the treatment group after administration of each dose,

C_e_: PK/PD parameters, AUC_24h_/MIC_99_, C_max_/MIC_99_, %T >MIC_99_,

EC_50_: value of the PK/PD parameter to reach half of E_max_,

N: Hill coefficient, the slope of the PK/PD parameter, and E curves.

The fitting relationships between PK/PD parameters and E were expressed by the correlation coefficient (R^2^). The greater the value, the better was the fitting. PK/PD parameters were calculated to make the bacterial population decrease by 0 log_10_ CFU/mL, 3 log_10_ CFU/mL, and 4 log_10_ CFU/mL.

## Results

### MIC, MIC_99_, and MPC

The MIC, MIC_99_, and MPC were 0.0625, 0.05, and 0.4 μg/mL, respectively.

### PK

**Figure 1:**
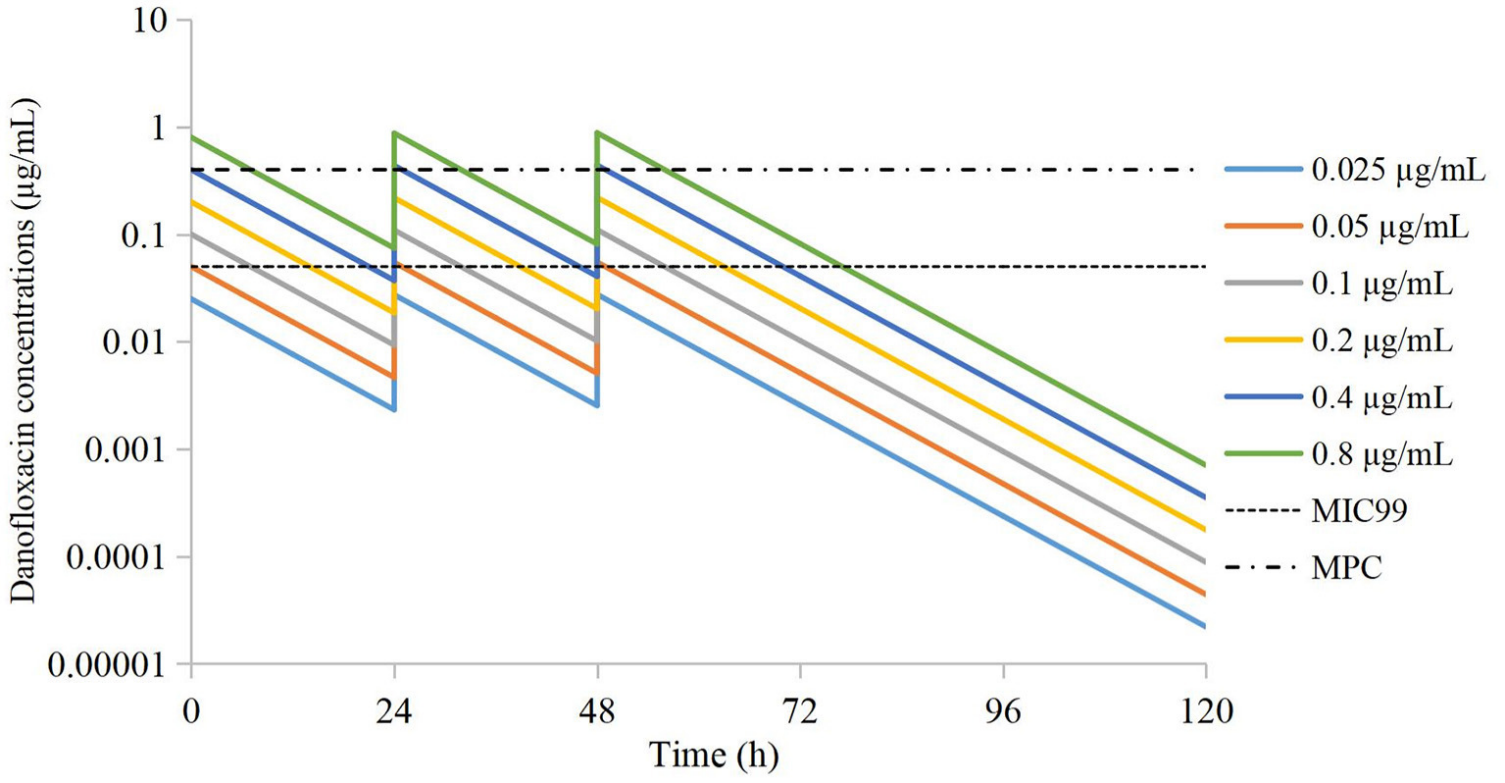
Simulated time-concentration curves of danofloxacin in the peristaltic pump after extrapolation.

According to Equation 1, the danofloxacin concentration at each time point was obtained by extrapolation. Concentration–time curves were drawn (Figure 1). The values of C_max_ and AUC_24h_ after administration of each dose were obtained using WinNonlin.

Drug concentrations were distributed in different parts of the MSW. The groups of 0.025 μg/mL and 0.05 μg/mL were located outside the MSW. The groups of 0.1 μg/mL and 0.2 μg/mL were located in the lower part of the MSW. The group of 0.4 μg/mL was located in the middle of the MSW. The group of 0.8 μg/mL group was located in the middle and upper parts of the MSW.

### *In vitro* dynamic kill curves

**Figure 2:**
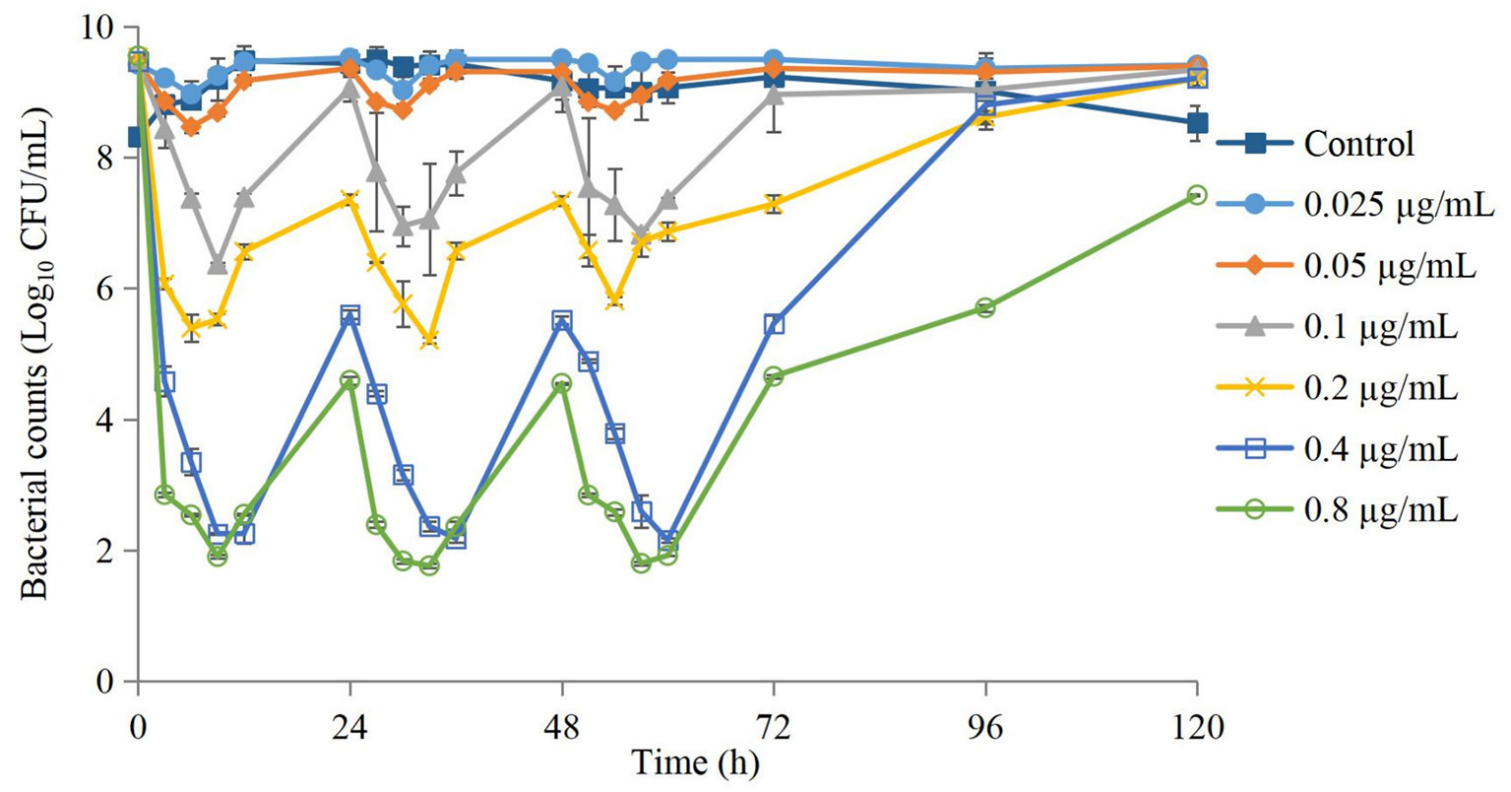
Time-kill curves of danofloxacin against *Actinobacillus pleuropneumoniae* after administration of different dosages. Values are the mean ±standard deviation (*n* = 3).

Kill curves at different dosing concentrations are shown in Figure 2. The antibacterial effect after each dose is shown in Table 1. The groups of 0.025 μg/mL and 0.05 μg/mL could produce a bacteriostatic effect. The group of 0.1 μg/mL could reach a bactericidal effect. The group of 0.2 μg/mL could reach an eradication effect.

**Table 1:** Values of the antibacterial effect (E) and PK/PD parameters of danofloxacin against *Actinobacillus pleuropneumoniae* after three-times administration.

**Dose (μg/mL)**	**AUC_24h_/MIC_99_ (h)**	**C_max_/MIC_99_**	**%T >MIC_99_ (%)**	**E (log_10_ CFU/mL)**
Control	0	0	0	1.16
	0	0	0	−0.27
	0	0	0	−0.17
0.025	0.50	4.73	0	−0.45
	0.55	5.17	0	−0.49
	0.55	5.21	0	−0.35
0.05	1.00	9.46	0	−0.99
	1.09	10.34	4.13	−0.63
	1.10	10.42	4.48	−0.58
0.1	2.00	18.92	29.59	−3.10
	2.19	20.67	33.33	−2.10
	2.20	20.84	33.64	−2.26
0.2	4.00	37.84	62.92	−4.14
	4.37	41.34	67.93	−2.15
	4.41	41.68	68.36	−1.53
0.4	8.00	75.68	92.42	−7.22
	8.74	82.69	94.93	−3.42
	8.81	83.36	95.14	−3.37
0.8	16.00	151.36	100.00	−7.65
	17.49	165.38	100.00	−2.84
	17.62	166.72	100.00	−2.75

AUC_24h_, 24-h area under concentration-time curve; C_max_, maximum concentration; MIC_99_, the minimum concentration that inhibits colony formation by 99%; %T > MIC_99_, the percentage of time that drug concentration remained above MIC_99_; Each dose was given thrice. From top to bottom corresponds to the antibacterial effect and PK/PD parameters after each dose.

**Figure 3:**
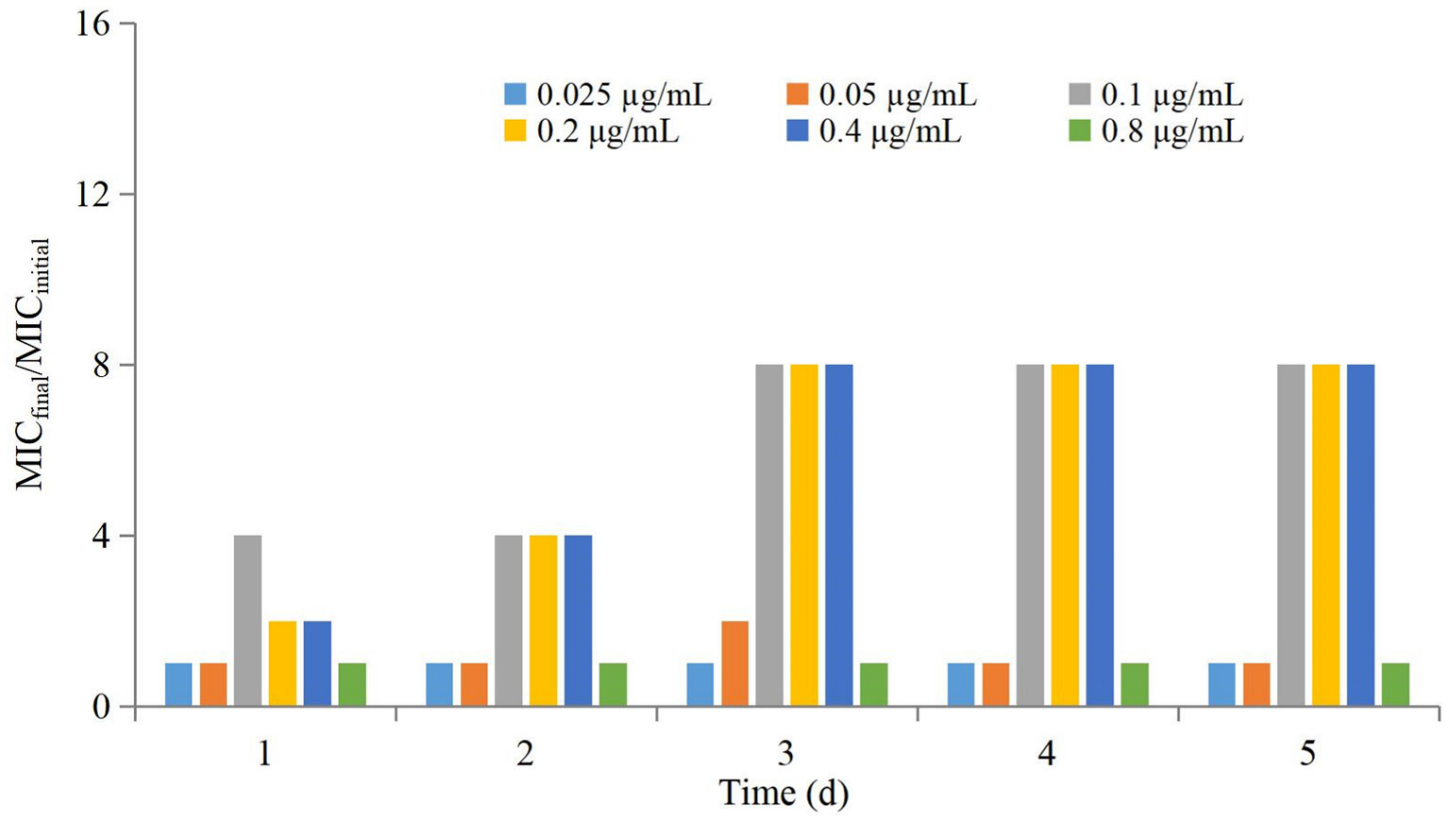
Values of MIC_final_/MIC_initial_ after drug administration for a different number of times.

The antimicrobial effect of three-times administration was not significantly different for the group of 0.025 μg/mL compared with that of the control group (Table 1). If the dose >0.025 μg/mL, then the reduction in the bacterial count after the first-time dose was significantly greater than that after the second-time dose and third-time dose. The higher the dose, the greater was the difference, but the difference between the second-time dose and third-time dose was not significant. Changes in the MIC at each dose are shown in Figure 3. The MIC of AP did not change significantly if the drug concentration was lower than MIC_99_ and higher than the MPC. If the drug concentration was in the middle of the MSW, then the MIC of AP was increased significantly if the frequency of drug administration increased (eightfold increase for the groups of 0.1, 0.2, and 0.4 μg/mL) and recovery to the initial value was observed in the group of 0.05 μg/mL after the final administration.

### PK/PD analysis

**Figure 4:**
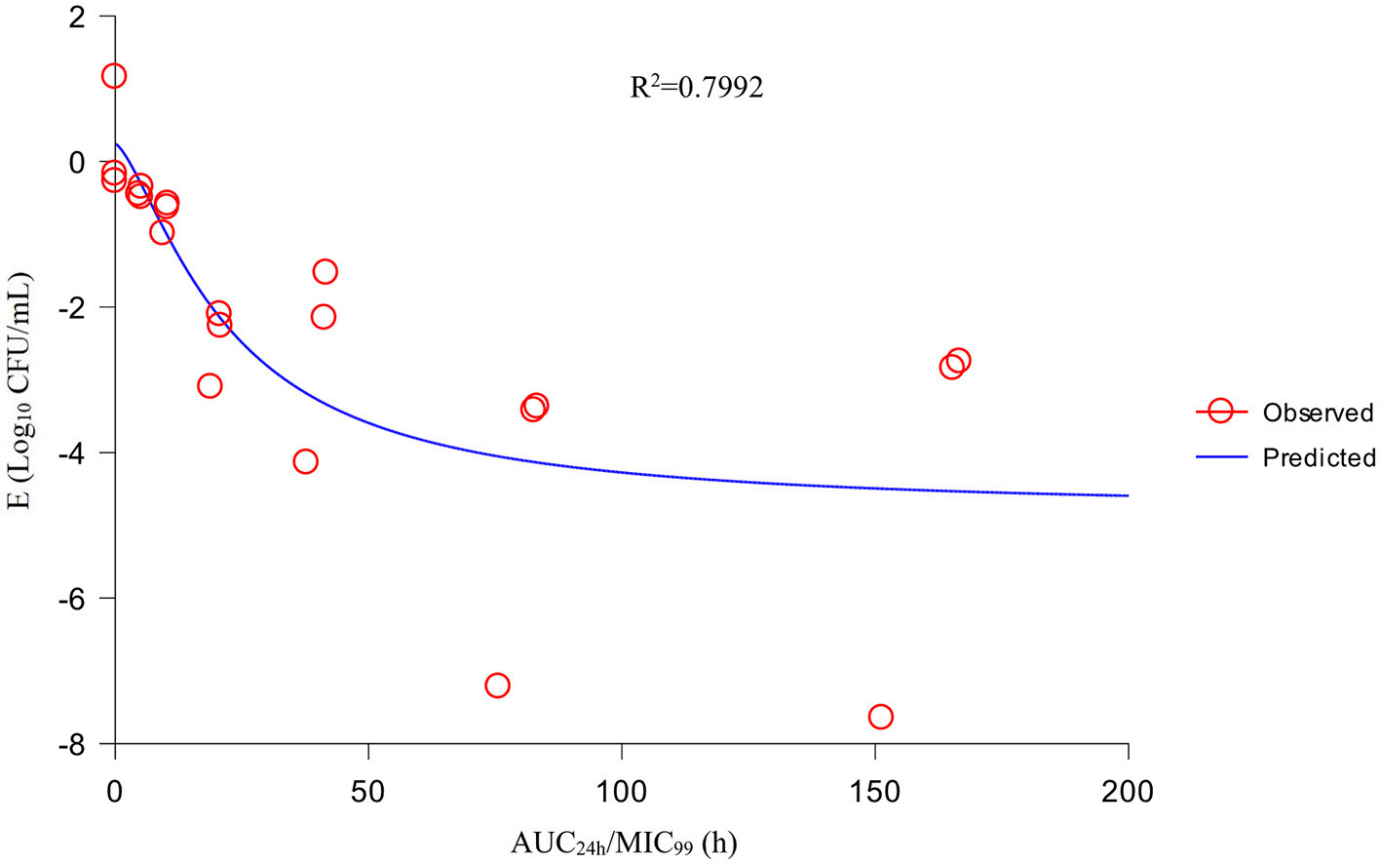
Fitting curve between AUC_24h_/MIC_99_ and the antibacterial effect.

**Figure 5:**
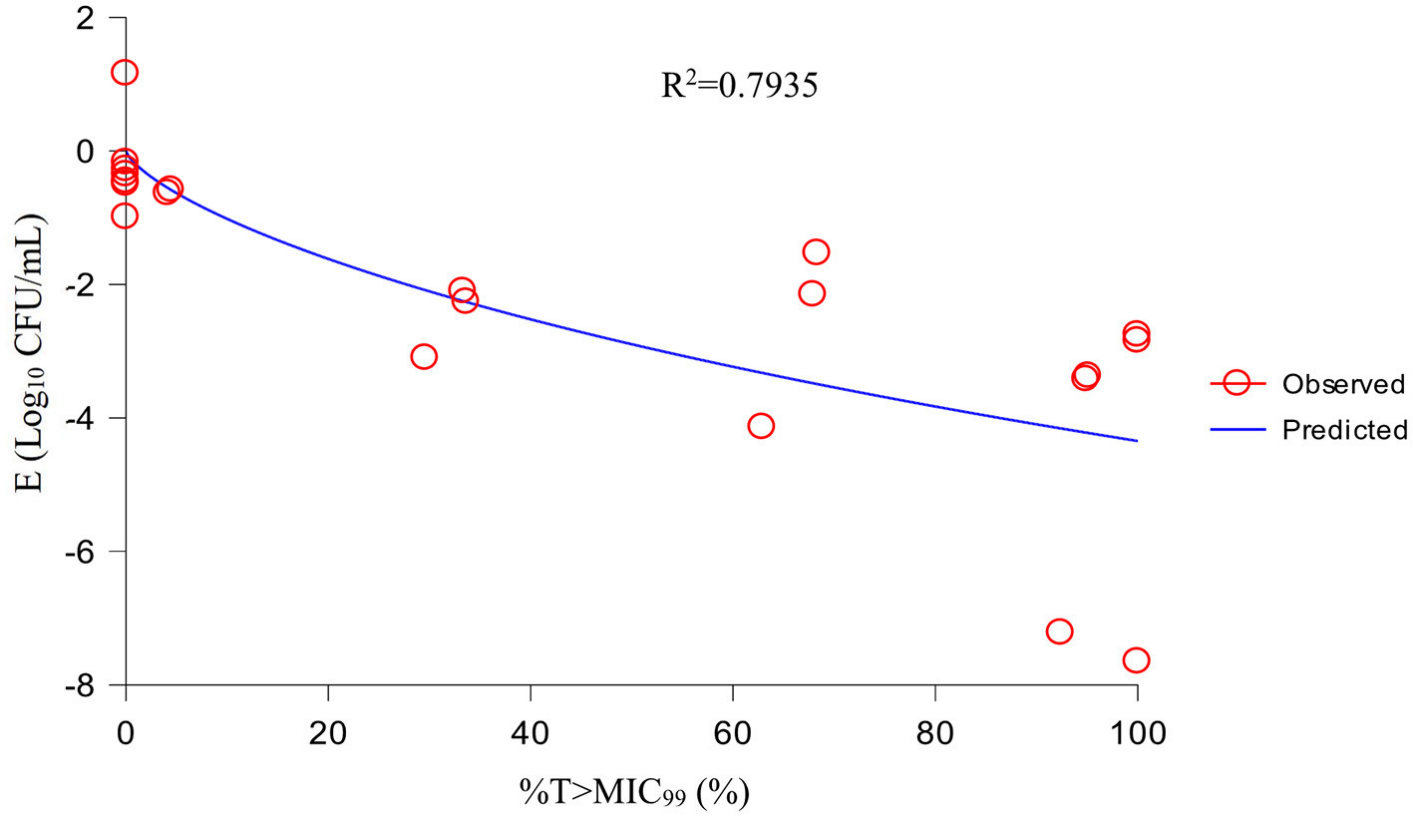
Fitting curve between %T >MIC_99_ and the antibacterial effect.

Using the sigmoid E_max_ model, AUC_24h_/MIC_99_ had the highest correlation with E (R^2^ = 0.7992) (Figure 4). R^2^ of %T >MIC_99_ with E was 0.7935 (Figure 5). The values of AUC_24h_/MIC_99_, C_max_/MIC_99_, %T > MIC_99_, and E are shown in Table 1. Therefore, AUC_24h_/MIC_99_ was selected as the PK/PD parameter to predict the corresponding E. The PK/PD parameters and AUC_24h_/MIC_99_ values for different antibacterial effects were obtained (Table 2). The predicted values of AUC_24h_/MIC_99_ to produce a bacteriostatic effect, bactericidal effect, and eradication effect were 2.68 h, 33.67 h, and 71.58 h, respectively.

**Table 2:** Values of PK/PD parameters and AUC_24h_/MIC_99_ to achieve different antibacterial effects.

**PK/PD parameter**	**Value**
E_max_ (Log_10_ CFU/mL)	0.24
EC_50_ (h)	22.30
E_0_ (Log_10_ CFU/mL)	−4.81
Slop (N)	1.42
AUC_24h_/MIC_99_ for bacteriostatic effect (h)	2.68
AUC_24h_/MIC_99_ for bactericidal effect (h)	33.67
AUC_24h_/MIC_99_ for eradication effect (h)	71.58

E_max_, change in the bacterial count in the control group after administration of each dose; E_0_, maximum change in the bacterial count in the treatment group after administration of each dose; EC_50_, value of the PK/PD parameter to reach half of E_max_; N, Hill coefficient, the slope of the PK/PD parameter and E curves.

## Discussion

Danofloxacin is a third-generation fluoroquinolone used solely in animals. It has a wide range of antibacterial activities. The *ex vivo* PK/PD of danofloxacin have been studied in ruminants (e.g., sheep, goats, cattle, camels) using tissue-cage infection models, but reports for bacteria that infect pigs are scarce. Although those *ex vivo* studies reflected the interaction between the host, drugs, and bacteria comprehensively, the drug concentrations were constant. Therefore, a new model is needed to ascertain the influence of dynamic drug concentrations on pathogens.

Previously, we studied the *in vivo* PK/PD integration of danofloxacin against AP using a tissue-cage infection model. However, the targets of AP are the lungs and blood, so differences exist between tissue fluid and lungs. Considering the high cost and fatality rate using an animal-infection model, establishment of an *in vitro* infection model to simulate infection of target organs is necessary and valuable.

The peristaltic-pump model can be employed to simulate the dynamic changes in drug concentration and bacterial population in the host in real-time. This strategy provides important support for simulating *in vivo* PK/PD integration (especially for simulation of difficult-to-obtain target organs). Therefore, we established a peristaltic-pump infection model to study the MSW-based PK/PD integration of danofloxacin against AP *in vitro* for preventing the emergence and spread of drug-resistant mutant bacteria. In the present study, the PK parameters of danofloxacin in pig blood were referenced with results reported previously (t_1/2_ = 7.28 ± 1.10 h) (27). We set t_1/2_ at 7 h after comprehensive consideration of the deviation of different dosing methods and reagents.

Kill curves revealed a marked difference in the antibacterial effect among three-times administration at an identical dosing concentration. In particular, the antibacterial effect of one-time administration was obviously higher than that for two-times and three-times administration. Three main reasons could explain these results. First, the growth rate of mutant AP may be reduced to add its persistence against drugs. Second, a sub-inhibitory concentration of danofloxacin could inhibit the growth of bacteria. Third, the drugs in the bacterial body have antibacterial activity. Therefore, bacteria need a long time to pump-out drugs. Hence, the rate of bacterial growth is reduced and bacterial counts cannot recover to that in the initial population.

The sensitivity of AP decreased if the danofloxacin concentration was between MIC_99_ and the MPC. These experimental results are consistent with those reported by other investigators (28–31). The main reason is that sensitive bacteria were the main subpopulation in the original population, but a few resistant subpopulations were present. These sensitive bacteria were killed gradually if the drug concentration was between MIC_99_ and the MPC. After multiple dosing, the resistant subpopulations grew gradually and became the main population that exhibited a higher MIC compared with that of the bacteria in the original population. Therefore, drug concentrations located in the lower part of the MSW should be avoided if dosage regimens are being designed.

If selecting drugs for the treatment of bacterial infections, PK/PD parameters are used often to evaluate the clinical efficacy of antimicrobial agents to prevent the emergence and spread of drug-resistant bacteria (32). For fluoroquinolones, the best-fitting PK/PD parameter related to the antibacterial effect is AUC_24h_/MIC (33). We also analyzed the relationship between C_max_/MIC_99_, %T >MIC_99_, and AUC_24h_/MIC_99_, and the antibacterial effect. We discovered that AUC_24h_/MIC_99_ and C_max_/MIC_99_ were correlated more strongly with antibacterial activity (*R*^2^ = 0.7992 and 0.7991, respectively) compared with %T >MIC_99_ (*R*^2^ = 0.7935). Hence, we applied AUC_24h_/MIC_99_ to analyze the PK/PD parameters between E and calculate the required values of AUC_24h_/MIC_99_ to achieve different antibacterial efficacy. The predicted values of AUC_24h_/MIC_99_ to produce a bacteriostatic effect, bactericidal effect, and eradication effect were 2.68, 33.67, and 71.58 h, respectively.

Few PK/PD studies of danofloxacin against pathogenic bacteria in pigs have been conducted. The *ex vivo* PK/PD of danofloxacin against *Pasteurella multocida* and *Haemophilus parasuis* in piglet serum were studied by Li et al. (34). The mean values of AUC_24h_/MIC to produce a bacteriostatic effect and bactericidal effect were 32 h and 49.8 h for *P. multocida*, whereas they were 14.6 h and 37.8 h for *H. parasuis*, respectively. Yang et al. (35) studied the *ex vivo* PK/PD integration of danofloxacin against *Escherichia coli* in piglet ileum using ultrafiltration probes. The mean values of AUC_24h_/MIC for ileum ultrafiltrates that achieved a bacteriostatic effect, bactericidal effect, and eradication effect were 99.85, 155.57, and 218.02 h, respectively.

## Conclusions and recommendations

We established an *in vitro* peristaltic-pump infection model to simulate the dynamic changes in danofloxacin concentrations in pig plasma. We obtained real-time and continuous PK data and PD data simultaneously. AUC_24h_/MIC_99_ was the best-fitting PK/PD index for the antibacterial effect. The predicted values of AUC_24h_/MIC_99_ to produce a bacteriostatic effect, bactericidal effect, and eradication effect were 2.68, 33.67, and 71.58 h, respectively. These results may provide a valuable reference for application of danofloxacin in the treatment of AP infection.

## Data availability statement

The raw data supporting the conclusions of this article will be made available by the authors, without undue reservation.

## Author contributions

LZ and HW contributed to the methodology, software use, validation, data analysis, writing, and project administration. YiB, YuB, and LW contributed to study supervision, manuscript revision, and funding acquisition. All authors read and approved the final version of manuscript.
